# Supplementary material for: The cAMP signaling pathway regulates Epe1 protein levels and heterochromatin assembly
Source: PLoS Genet. 2022 Feb 16;18(2):e1010049. doi: 10.1371/journal.pgen.1010049 (PMC8887748; doi:10.1371/journal.pgen.1010049)
Supplement: Attachment — Submitted filename: Response to reviewers.docx [file pgen.1010049.s008.docx]

Reviewer #1: The paper by Jia and colleagues identifies the cAMP-PKA signaling pathway as a regulator of translation efficiency of Epe1, a putative H3K9me demethylase that negatively regulates heterochromatin spreading. Thus, the authors identify a link between a nutritional-sensitive pathway and he formation of heterochromatin.
The authors employ the S. pombe deletion library to isolate mutant strains that affect heterochromatin at the centromere. The query strain contains a mild overexpression of Epe1 (nmt41-Epe1) and a reporter gene at the centromeric heterochromatin (otr::ura4+). They identify 5 deletion mutations in the cAMP/PKA signaling pathway. Analysis of the transcription levels of pericentric repeats (dh) suggests that loss of cAMP/PKA signaling affects heterochromatin formation in cells overexpressing Epe1 at a natural chromosomal context (Fig. 1-2E). The authors go on to show a correlation between intact cAMP/PKA signaling and the protein level of Epe1 (Fig. 2F). Using polysome profiling, the authors demonstrate a decrease in epe1+ mRNA in the polysome fractions in git3 mutant cells, suggesting that cAMP/PKA affects the translation efficiency of epe1+ mRNA. Furthermore, the authors show that the cAMP-PKA pathway affects the endogenous levels of Epe1 (Fig. 4A) and that git3 mutant cells, like epe1 mutant cells, affect heterochromatin spreading outside the mating type locus and increase H3K9me2 at heterochromatic islands. Consistent with the idea that cAMP/PKA affects Epe1 levels, the authors show that in low glucose, the protein levels of Epe1 are reduced and there is a slight increase in HE3K9me at heterochromatin islands.

The data is generally of high quality and the findings are novel and of interest to the field of chromatin biology. To my opinion, the main weakness of the article is the limited analysis of the effect of the cAMP-PKA signaling on wild type cells expressing endogenous levels of Epe1 (see comment 3 and 4 below).

The reviewer noted that our data “is generally of high quality” and “the findings are novel and of interest to the field of chromatin biology”. We appreciate the reviewer’s encouraging comments. Below we addressed the specific concerns. Importantly, we added new data to examine the effects of the cAMP signaling on heterochromatin in wild type cells.

Concerns:

1. Fig. 2E. The authors suggest that "cgs2Δ, cgs1Δ, or the addition of cAMP have little effects on dh transcript". Statistical analysis is missing.

We have included statistical analysis on Fig. 2E.

2. Fig. 3C. It is not clear to me how the experiment was performed, since the level of Epe1 at time 0 is similar in WT and git3 cells (but Epe1 levels are reduced in git3 cells, as shown in Fig. 3B).

In Figure 3C, we used different exposures of HA-Epe1 western for WT and *git3∆* cells, so that the reader can have a better visual comparison of degradation rate. We added this description in the figure legends.

3. What is the effect of deletion of git3 on the steady state levels of endogenous Epe1? And/or:

The 3HA-Epe1 expressing strains contain a N-terminus 3HA tag inserted at the endogenous *epe1^+^* chromosomal location, with its endogenous promoter, 5’- and 3’-UTRs. Therefore, Fig. 4A shows the effects of *git3∆* on endogenous Epe1.

4. What is the effect of deletion of git3 on translation efficiency of epe1+ mRNA in wild type cells?

We performed additional ribosome profile experiment (new Fig. 4D and 4E), which shows that endogenous *epe1^+^* mRNA translation efficiency is also reduced in *git3∆* cells.

5. There is relatively little data to show that low glucose affects heterochromatin formation (Fig. 4 E, G). The authors may consider adding the effect of mutating cgs2 on the levels of Epe1 in low glucose (Fig. 4E). In addition, does low glucose affect heterochromatin spreading outside the mating type locus (similar to the effect of the epe1 mutation, Fig. 4C)?

Given the similar effects of *cgs1∆* and *cgs2∆*, we examined Epe1 protein levels in *cgs1∆* cells in low glucose conditions (new Figure S5). Consistent with our hypothesis, in low glucose conditions, Epe1 protein levels are higher in *cgs1∆* cells.

The time frame of the low glucose treatment is not compatible with dilution analysis. We therefore performed qRT-PCR analysis of the expression *SacI::ade6^+^* as an indicator of heterochromatin spreading at the mating-type region (new Figure 6C). The result shows that *ade6* expression in wild type cells is indeed reduced in low glucose condition, consistent with increased heterochromatin spreading.

6. The manuscript should be carefully edited for typos.

We have further checked our manuscript and tried our best to reduce typos.

Reviewer #2: Bao et al. use a genetic screen to search for suppressors of Epe1 overexpression-mediated loss of heterochromatin and gene silencing. Epe1 is a JmjC domain anti-silencing factor that promotes histone H3K9 demethylation, and plays a major role in preventing inappropriate H3K9me3 spreading and epigenetic maintenance. The authors cross nmt41-epe1 overexpressing cells to the S. pombe deletion library for non-essential genes to search for genes that may regulate Epe1 expression or function. Their screen uncovers multiple genes in the cAMP signaling pathway and they go on to demonstrate that cAMP signaling is required for high level expression of both nmt41-driven and endogenous Epe1. They further show that cAMP signaling acts through the Pka1 kinase to promote efficient translation of epe1+ mRNA. They conclude that the cAMP pathway regulates heterochromatin formation in response to changes in nutritional signals. The experiments are straightforward, well-executed, and support the main conclusion of the paper. Altogether, the study provides new insight into how changes in signaling pathways linked to growth conditions and metabolism regulate the expression of the JmjC domain protein Epe1 and provides evidence that this regulation takes place at the level of translation. The authors should consider the following points.

The reviewer noted that “the experiments are straightforward, well-executed, and support the main conclusion of the paper”. We thank the reviewer for the encouraging comments. Below we have addressed the specific concerns raised.

1. Is the regulation of translation by cAMP signaling general or specific to epe1+. The only controls the authors provide, nmt1+ and act1+ polysome associations (Figure 3D), seem to indicate that in git3-delta cells, polysome association of both these control mRNAs is reduced. Both nmt1+ and act1+ are highly abundant mRNAs. Have the authors tested the effect of deleting git3 on a few other mRNAs that are lowly expressed? This would be a valuable addition to the data in Figure 3.

We performed additional qRT-PCR analysis of another lowly expressed mRNAs, *clr4^+^*. The new Figure S3 shows that although translation efficiency of *clr4^+^* mRNA is slightly reduced, there is no change in Clr4 protein levels. Therefore, mutations of cAMP signaling pathway seem to have a general reduction on mRNA translation, but *epe1^+^* mRNA is much more sensitive to these mutations than other mRNAs.

2. Epe1 has also been shown to play a critical role in DNA sequence-independent epigenetic inheritance of H3K9me and heterochromatin (PMID: 25831549, PMID: 25838386). It would be great to know whether attenuation of cAMP signaling would allow epigenetic inheritance of heterochromatin in epe1+ cells.

We performed experiment to examine the impact of *git3∆* on epigenetic inheritance of an ectopic heterochromatin (established by TetR-Clr4-SET at tetO-GFP) as suggest by the reviewer. However, the result is inconclusive because *git3∆* unexpectedly affects the silencing of the TetO-GFP reporter before the addition of tetracycline (new Fig. S4B).

We therefore used a different system to measure heterochromatin inheritance. Loss of Epe1 bypasses RNAi for pericentric heterochromatin formation, which is also attributed to DNA sequence-independent heterochromatin inheritance. We found that *git3∆* also rescues *ago1∆* phenotype in the silencing of *otr::ura4^+^* and TBZ sensitivity, consistent with the idea that *git3∆* phenocopies *epe1∆* (new Fig. 5C)

3. The authors present ChIP-seq data showing increased H3K9me2 levels at the mating type locus when cells are grown in low glucose. Does LG affect H3K9me2 spreading at other loci or the silencing of the Sac1::ade6+ reporter? Does low LG allow sequence-independent heterochromatin inheritance?

The new Figure 6D shows H3K9me2 levels at individual heterochromatin islands and the major ones consistently show increases of H3K9me2 levels.

Since the short time frame of low glucose experiment is not compatible with dilution analysis, we performed qRT-PCR analysis of the expression *SacI::ade6^+^* as an indicator of heterochromatin spreading at the mating-type region. The result shows that *ade6^+^* expression in wild type cells is reduced in low glucose condition, indicating increased heterochromatin spreading (new Fig. 6C).

We tested the effects of LG on heterochromatin inheritance using the tetO-GFP tetR-Clr4-SET system. Although the data seems to suggest that low glucose condition slows down heterochromatin decay in wild type cells, we note that the reporter is slightly desilenced before tetracycline addition and that cells grow slower in LG, making the results difficult to interpret (new Fig. S4C).

Was the ChIP-seq in Fig. 4G performed on multiple clones to ensure that the authors are not looking at small sample to sample variations in H3K9me2 levels?

The ChIP-seq result shown is the average of two experiments using the same clone. For each replicate, we performed YEA and LG together. The two replicates were performed separately and the results show good reproducibility. Therefore, the changes are unlikely due to sample-to-sample variations. In our ChIP-seq analyses, H3K9me2 levels at major heterochromatin islands are consistent, and larger variations occur mainly at small islands. New Fig. 6D shows that the top 5 heterochromatin islands all clearly show increases of H3K9me2.

Minor
-Does the 3X HA tag affect Epe1 function?

The 3xHA-epe1 strain has only minor effects on silencing of *sacI::ade6^+^* compared with *epe1∆*, suggesting that the tagged version of Epe1 is mostly functional.

Reviewer #3: General comments:
Environmental changes lead to transcriptional changes. However, the role of chromatin in this process is not well understood. In this study, the authors devise a clever genetic screen to explore how the chromatin modifier, Epe1, regulates a transcriptionally repressive chromatin mark (H3K9me2) in Schizosaccharomyces pombe. Surprisingly, the authors find components of the conserved cAMP signaling pathway regulate the activity of Epe1. Using the powerful genetics of S. pombe, the authors demonstrate that cAMP signaling has an important role in the efficient translation of epe1 mRNA, maintenance of Epe1 levels, and regulation of chromatin landscape in response to environmental changes. In summary, the authors present a very interesting study that demonstrates a novel link between environmental signaling and chromatin regulation.

The manuscript is within the scope of PLOS Genetics.

We thank the reviewer for the encouraging comments. Below we have addressed the specific concerns raised.

The authors should add line and page numbers to aid the review process.

We added line numbers in the revised manuscript.

The authors should check comma usage throughout the manuscript and ensure manufacturer information is provided and consistent for all commercial products.

We further checked comma usage and that manufacture information for all commercial products are included.

Major comments:
The third paragraph in the discussion walks through experiments that were conducted during the study. These experiments are very interesting and a logical next step for this work. However, the authors discuss data not included in the manuscript due, at least partially, to lack of robustness (mass spec data, i.e., “We could not rule out the presence of additional sites because the peptide coverage was not 100% and the phosphorylation is weak.”). Results should not be presented in the discussion particularly in the absence of data. If results are robust enough to discuss, they (and the underlying data) should be presented in the results section.

We added the data in new Figure S1 and described the findings in the results section.

In materials and methods, the authors state how peaks were called (MACS2) and heatmaps were generated (deeptools2); however, it is unclear that this data is presented in this manuscript. It would be interesting to see additional results from the ChIP-seq data, i.e., how individual heterochromatin islands change or if new H3K9me2 peaks were observed. If these results were assessed, are robust, and provide additional evidence in support of this study, they should be presented in the results section. Otherwise, materials and methods should be changed accordingly.

We included new Figure 6D, which summarizes the ChIP-seq results of individual heterochromatin islands.

Additional comments:
Abstract:
The following sentence overstates the impact of this study and should be rewritten: “These results provide a mechanism for the regulation of heterochromatin in response to changes in nutritional conditions.”

We changed this sentence to “These results reveal the mechanism by which the cAMP signaling pathway regulates heterochromatin landscape in fission yeast”.

Results:

The following sentence should be rewritten for clarity: “In the presence of cAMP, Cgs1 dissociates from Pka1, leading to Pka1 translocation to the nucleus and its activation to phosphorylates its substrates.”

We changed this sentence to “In the presence of cAMP, Cgs1 dissociates from Pka1. Pka1 then translocates to the nucleus and phosphorylates its substrates.”

Based on the data presented and inherent limitations with ChIP-seq, it is difficult to claim that “average H3K9me2 levels increase” in low glucose conditions (a quantitative ChIP-seq approach could provide support for the claim). A claim that “average H3K9me2 levels appear to increase” is reasonable. Additionally, presenting data for individual heterochromatin islands could provide clearer results. Of particular interest would be ChIP-seq data for the ssm4 and mei4 loci, which are quite different in relevant mutants in ChIP-qPCR experiments. See the following sentence: “ChIP-seq analyses show that heterochromatin islands positions are maintained and average H3K9me2 levels increase in cells grown in a low glucose medium for 6 hours compared to cells grown in the rich medium (Fig. 4G), consistent with the reduction of Epe1 levels.”

We changed the claim to “H3K9me2 levels at many heterochromatin islands appear to increase” as suggested by the reviewer.

We provided analysis of individual heterochromatin islands, including *mei4^+^* and *ssm4^+^*, in new Figure 6D.

Materials and Methods:
Media (plural) should be replaced with medium (singular) in the following sentence and throughout the manuscript as needed: “Yeasts were grown in EMM media or YEA media.” Additionally, the authors should define abbreviated media and provide recipes or references for media used in this study.

We corrected the grammar mistake, added full name of media, and provided catalogue number for EMM or recipe for YEA.

The authors should provide additional information on the source of the tubulin antibody used in this study. As described, these experiments could not be replicated. See the following sentence: “The membrane was blotted with antibodies against Tubulin (gift) and HA (sc7392, Santa Cruz).”

We provided the source of Tubulin antibody and reference.

The authors should clarify the meaning of “25 OD” in the following sentence: “25 OD of yeast cells were lysed using a Fast Prep machine in polysome lysis buffer (20 mM Tris- HCl pH 7.5, 10 mM magnesium chloride, 50 mM potassium chloride, 10 μg/ml cycloheximide, 1 mM PMSF, 1x HaltTM protease and phosphatase inhibitor cocktail (ThermoFisher Scientific, 78442).” Additionally, the first parenthesis is not closed.

We changed “25 OD cells” to “Approximately 5x10^8^ yeast cells” and added the parenthesis.

Figures and Tables:
Figure 1C: Some protein names in the figure are in bold. These appear to be components that were identified in the genetic screen. The authors should describe what is represented by proteins in bold in the figure legend.

We added description in figure legend that the factor in bold are those identified in our genetic screen.

Figure 4D: The authors state that three loci were tested. However, results for only two loci are presented (and mentioned in results). See the following sentence: “ChIP analyses of H3K9me2 levels at three heterochromatin island loci.”

We corrected this mistake.

Figure 4G: The authors should provide additional information pertaining to what is shown in panel G.

We changed Figure 4G to the new Figure 6D, which showed individual heterochromatin islands rather than the average.
